# TSCNet: transformer-simplicial convolutional network for functional brain network classification

**DOI:** 10.3389/fmed.2026.1974555

**Published:** 2026-10-28

**Authors:** Libing Bai, Guorong Zhang, Liping Feng, Lu Shen, Bin Zhou, Feng Hu

**Affiliations:** 1 Department of Artificial Intelligence and Data Science, XinZhou Normal University, Xinzhou, China; 2 Computer College, Qinghai Normal University, Xining, China; 3 The State Key Laboratory of Tibetan Intelligence, Qinghai Normal University, Xining, China

**Keywords:** functional brain network, higher-order network, simplicial complex, simplicial convolutional network, transformer

## Abstract

Functional brain network modeling provides an important tool for analyzing disease-related abnormalities in functional connectivity. Conventional graph models, however, treat nodes and edges as their basic objects and mainly encode pairwise relationships between brain regions, making it difficult to explicitly represent higher-order topological units jointly formed by multiple regions. We therefore propose TSCNet, a functional brain network classification framework that integrates simplicial convolution with a Transformer. TSCNet first maps multi-site-harmonized functional connectivity matrices to hierarchical simplicial complexes containing 0-, 1-, and 2-simplices. Hodge-Laplacian-driven simplicial convolutions then extract local topological features separately at each order. These order-specific features are projected into a shared representation space and passed to a Transformer to model global dependencies across simplices and brain regions. We evaluated TSCNet on three public fMRI datasets, ABIDE, ADHD-200, and Haxby, using stratified 10-fold cross-validation repeated five times. The complete model achieved classification accuracies of 81.23% on ABIDE and 82.42% on ADHD-200. Removing either the SCNN or Transformer component reduced performance, indicating that local higher-order topology and global information integration provide complementary information. Parameter-sensitivity analyses and topological-pattern visualizations further characterized the model’s responses to simplex order and construction threshold. TSCNet provides a framework for jointly analyzing local higher-order topology and whole-brain dependencies, although its generalization to unseen scanners and the clinical specificity of the identified higher-order patterns require validation in independent cohorts.

## 1 Introduction

Functional magnetic resonance imaging (fMRI) noninvasively records brain activity and provides an important basis for studying cognitive processes and disease-related abnormalities at the network level. Functional brain networks typically represent brain regions as nodes and statistical associations between regional time series as functional connections, thereby yielding quantitative representations for disease classification and mechanistic analysis (1–4). Abnormal connectivity within the default mode, social cognition, and executive control networks has received sustained attention in studies of autism spectrum disorder (ASD) and attention-deficit/hyperactivity disorder (ADHD) (4–7). Extracting stable and interpretable brain-network features from multi-site fMRI data is therefore a central challenge in computer-aided brain disorder classification.

Conventional graph models represent a brain network as G=(V,E), where each edge explicitly describes only a pairwise relationship between two brain regions. Even when three nodes are mutually connected, the triangle is not explicitly distinguished as a two-dimensional unit that can carry features and participate in computation. A simplicial complex consists of simplices that satisfy the closure property: each k-simplex contains all of its lower-dimensional faces. It can therefore represent nodes, edges, and triangles as topological units of different orders within a unified structure (8, 9). This hierarchy does not by itself establish irreducible multivariate causality in the brain. It does, however, allow the model to treat joint topological patterns shared by multiple regions as distinct analytical objects and to propagate information across orders, extending representations learned only on nodes or edges.

Previous studies have extended functional brain network representations through graph models, hypergraphs, and topological deep learning. GCNs, GATs, and brain-network-specific graph models improve nonlinear aggregation of node or connection features, but their computational objects remain largely based on pairwise adjacency (10–16). Hypergraphs encode sets of brain regions with hyperedges and have been applied to functional connectivity analysis and brain disorder diagnosis (17–21). Simplicial and semi-simplicial learning further define signals on topological units of different orders for task decoding, individual identification, and brain-activity analysis (22–28). Existing simplicial convolutions effectively aggregate local topology through lower and upper adjacency, but a limited number of layers may not directly capture dependencies between distant regions or across functional networks. Global attention alone, in contrast, lacks explicit order and boundary constraints.

To address these limitations, we propose TSCNet, which combines the local topological inductive bias of simplicial convolution with the global attention of a Transformer. The model converts neuroCombat-harmonized functional connectivity matrices into topological distances and constructs simplicial complexes up to order 2 using a filtration threshold. It then uses the Hodge Laplacian at each order as a propagation operator to extract local features from 0-, 1-, and 2-simplices. The order-specific outputs are projected into a shared embedding space, and multi-head self-attention estimates global dependencies for classification. Simplicial convolution preserves topological units and local associations at different orders, whereas the Transformer expands the range of information exchange. Ablation experiments assess their complementarity.

The main contributions of this study are as follows:

1. We propose TSCNet for fMRI-based functional brain network classification. The model jointly learns local topological features on 0-, 1-, and 2-simplices and global dependencies, providing an implementable route beyond purely pairwise connectivity representations.
2. We design a feature flow aligned with the simplicial hierarchy. Hodge Laplacians drive order-specific simplicial convolutions, after which features from each order are projected into a shared embedding space and aggregated by a Transformer. This design separates local topological constraints from global attention-based fusion.
3. We evaluate TSCNet on three public fMRI datasets, ABIDE, ADHD-200, and Haxby. Module ablations and sensitivity analyses of the construction threshold, embedding dimension, simplex order, and network depth examine how individual design choices relate to performance.
4. We visualize 1-simplex and 2-simplex patterns separately to compare pairwise connections with candidate joint topological patterns involving three brain regions. Their disease specificity and clinical associations are treated as hypotheses requiring independent validation rather than as established biomarkers.

## 2 Related work and theoretical background

### 2.1 Graph and higher-order representations of functional brain networks

Traditional functional brain networks represent brain regions as nodes and statistical functional connections as edges, and use graph-theoretic measures or graph neural networks for disease classification (1, 2, 10–13). BrainNetCNN, FBNetGen, and Brain Network Transformer enhance pairwise connectivity representations through connectivity-matrix convolution, task-aware network generation, and global attention, respectively (14–16). Hypergraphs extend this representation by using a hyperedge to encode a set of brain regions; recent hypergraph foundation models, cross-template hypergraph Transformers, and higher-order pattern methods have also demonstrated the potential of higher-order structures for brain disorder analysis (19–21). Unlike an arbitrary hyperedge, a simplicial complex explicitly preserves face relations among structures of different orders, allowing nodes, edges, and triangles to serve as distinct signal domains.

Higher-order connectomics has applied local topological features to task decoding, individual identification, and disease-related brain-network analysis (22, 24). Recent work on semi-simplicial learning has further examined the expressive value of directed higher-order patterns for brain-activity decoding (23). These studies show that higher-order topology offers analytical objects beyond pairwise graphs, but they do not imply that simplicial complexes are inherently superior to graphs or hypergraphs for every task. In this study, simplicial convolution preserves hierarchical topology, the Transformer extends the range of global interactions, and ablation and sensitivity analyses test whether this combination is effective.

Given a finite set of vertices V, a k-simplex $S_{k}$ is a subset of V with cardinality k+1. A face of $S_{k}$ is a subset of $S_{k}$ with cardinality k, so $S_{k}$ has k+1 faces. The coplanarity of $S_{k}$ is a k+1 simplex containing $S_{k}$. The SC is obtained by gluing together simplexes $S_{k}$ of different dimensions. For any k-simplex $S_{k}$ in SC, any subset of $S_{k}$ must also be in SC. The dimension of SC is the maximum dimension of any simplex $S_{k}$ in it, denoted as k (25).

Generally, the boundary matrix $B_{k}$ represents the relationship between the k-simplex $S_{k}$ and the k−1-simplex $S_{k-1}$. The rows of $B_{k}$ represent the k-simplex, and the columns of $B_{k}$ represent the k−1-simplex. For example, $B_{1}$ represents the adjacency relationship between node and edge, and $B_{2}$ represents the boundary relationship between edge and triangle (9). For a simplex $S_{k-1}\subset S_{k}$, given a reference direction, $S_{k-1}∼S_{k}$ means that the k−1-simplex and the k-simplex have the same direction. Conversely, $S_{k-1}≁S_{k}$ means that the k−1-simplex and the k-simplex have different directions. $[B_{k}{]}_{i,j}$ is represented as Equation 1:

$$[B_{k}{]}_{i,j}=\left\{ \begin{array}{cc} 0\; & S_{k-1,i}⊄S_{k,j}\;\\ 1\; & S_{k-1,i}\subset S_{k,j}∥S_{k-1,i}∼S_{k,j}\;\\ -1\; & S_{k-1,i}\subset S_{k,j}∥S_{k-1,i}≁S_{k,j}\; \end{array}\right.,$$ (1)

In functional brain network, the 0-simplex corresponds to a single vertex (brain region), the 1-simplex corresponds to an edge formed by two vertices (paired connections between brain regions), the 2-simplex corresponds to a triangular structure formed by three vertices (three-brain region group association), and so on. The k-simplex can characterize the higher-order cooperative relationships of k+1 brain regions. Figure 1 shows an example of a simplical complex and its boundary matrix representation.

**Figure 1:**
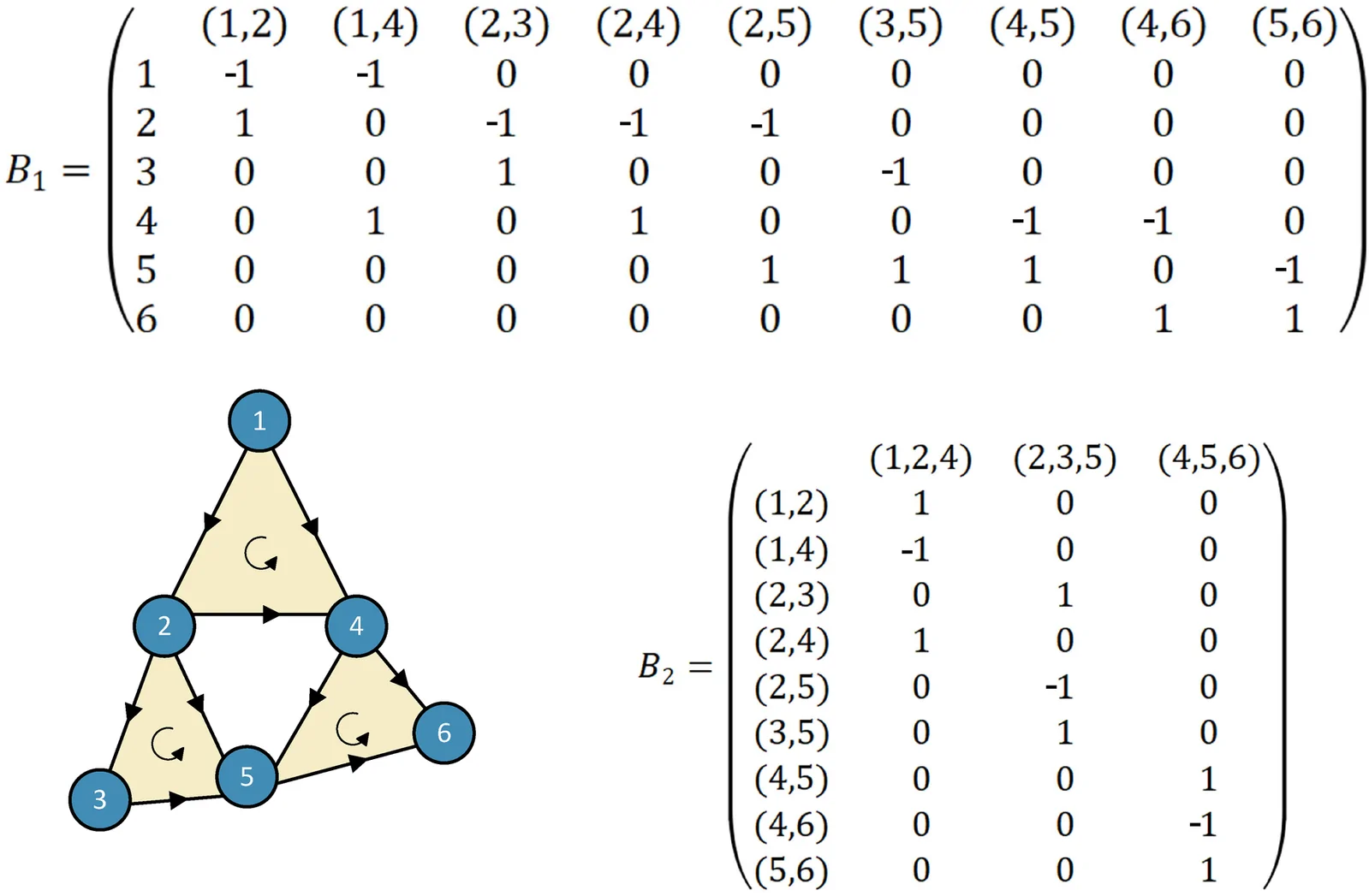
Simplicial complex representation.

### 2.2 Hodge Laplacian matrix

The Hodge Laplacian matrix (25) is a core mathematical tool for modeling high-order topological relationships using simplicial convolution, and its construction logic strictly follows the hierarchical structure of the simplicial complexes. For any k-dimensional simplex, the k-order Hodge Laplacian matrix $L_{k}$ can be expressed as Equation 2:

$$L_{k}=B_{k}^{T}B_{k}+B_{k+1}B_{k+1}^{T},$$ (2)

where $B_{k}\in R^{N_{k-1}\times N_{k}}$ is the k-order simplex boundary matrix, $N_{k}$ represents the number of k-simplex, $N_{k-1}$ represents the number of k−1-simplex, and its element $B_{k}$ is used to quantify the inclusion relationship between the j-th k-simplex and the i-th k−1-simplex; while $B_{k+1}^{T}\in R^{N_{k}\times N_{k+1}}$ is the transpose of the k+1-order boundary matrix, corresponding to the topological connection between the k-simplex and the k+1-simplex that contains it.

### 2.3 Simplicial convolution and local topological learning

A simplicial convolutional neural network performs topology-aware propagation on signals defined over simplices of different orders. Its core operator is the Hodge Laplacian constructed from boundary matrices (25–27). Unlike conventional graph models that aggregate only over nodes or edges, this operator uses both lower and upper adjacency among simplices of the same order. Existing methods mainly focus on local propagation. Modeling long-range dependencies across brain regions and functional networks while preserving order and boundary constraints therefore remains a key challenge addressed in this study.

In specific convolutional operations, simplicial convolutional achieves topologically aware feature aggregation through the normalized Hodge Laplacian matrix $\tilde{L_{k}}$, avoiding feature scale bias caused by directly using the original matrix. Assuming the initial feature matrix of the k-simplex is $X_{k}\in R^{N_{k}\times C}$ (where $N_{k}$ represents the number of k-simplex and C represents the input feature dimension of a single simplex), the local feature aggregation process can be represented as Equation 3:

$$X_{k}^{\prime }=\sigma (\tilde{L_{k}}X_{k}W_{k}+b_{k}),$$ (3)

where $W_{k}\in R^{C\times C^{\prime }}$ is a learnable feature transformation weight matrix, responsible for mapping the input feature dimension from C to the output dimension $C^{\prime }$; $b_{k}\in R^{1\times C}$ is a bias term, used to adjust the feature distribution to improve the model’s fitting ability; σ(⋅) is a non-linear activation function (such as ReLU), which enhances the model’s ability to express complex high-order features by introducing non-linear transformation; and the final output $X_{k}^{\prime }\in R^{N_{k}\times C^{\prime }}$ is the k-simplex feature matrix after convolution.

### 2.4 Role of the transformer in brain networks

The Transformer establishes dependencies among all input units through self-attention and can therefore extend the receptive field of finite-depth local propagation (29). Brain Network Transformer, Graphormer, and recent graph Transformer foundation models for functional connectivity networks indicate that global attention is suitable for capturing long-range relationships in brain networks (14, 30, 31). Attention alone, however, does not automatically encode simplex order, face relations, or cross-order topological constraints. TSCNet therefore uses Hodge-Laplacian-driven simplicial convolution to extract local features at each order before passing the uniformly embedded multi-order representations to a Transformer.

Self-attention mechanisms aggregate global information by calculating the association weights between each element and all elements in the sequence. The calculation process consists of three steps:

1. Query, Key, and Value Generation: A linear transformation is performed on the input feature matrix $X\in R^{n\times d_{in}}$ (where n is the number of brain regions and $d_{in}$ is the feature dimension of the brain region) to generate query, key, and value matrices represented as Equation 4.
  $$\begin{array}{cc} & Q=X\times W_{Q}+b_{Q}\\ & K=X\times W_{K}+b_{K}\\ & V=X\times W_{V}+b_{V} \end{array},$$ (4)
  $W_{Q}$, $W_{K}$ and $W_{V}$ are learnable weight matrices, while $b_{Q}$, $b_{K}$ and $b_{V}$ are learnable bias vectors. All three participate in the training and optimization of the model to achieve accurate mapping of brain region features.
2. Attention score calculation: The association strength is calculated by the dot product of the query and the key, divided by $\sqrt{d_{k}}$ for scaling to avoid gradient vanishing, and then normalized by softmax to obtain the attention weights represented as Equation 5:
  $$\text{Attention}=\text{softmax}\left(\frac{QK^{T}}{\sqrt{d_{k}}}\right)V,$$ (5)
  where $QK^{T}\in R^{n\times d_{in}}$ is the association score matrix of the brain regions, and the softmax function normalizes along the rows so that the sum of the attention weights of each brain region is 1.
3. Multi-head attention fusion: To capture global dependencies across multiple perspectives, Transformer introduces multi-head attention, executing the attention mechanism in parallel h times, and then concatenating and linearly transforming the results represented as Equation 6:
  $$\text{MultiHead}(Q,K,V)=\text{Concat}({\text{Head}}_{1},\ldots ,{\text{Head}}_{h})\cdot W_{O},$$ (6)
  where ${\text{Head}}_{i}=\text{Attention}(Q\cdot W_{Qi},K\cdot W_{Ki},V\cdot W_{Vi})$ is the output of the i-th attention head, $W_{O}\in R^{h\cdot d_{k}\times d_{out}}$ is the output weight matrix, and $d_{out}$ is the final output feature dimension.

## 3 Method

### 3.1 Datasets

We evaluated TSCNet on three public fMRI datasets: resting-state ABIDE (32), resting-state ADHD-200 (33), and task-based Haxby (34). ABIDE was used for binary classification of ASD and healthy controls, ADHD-200 for binary classification of ADHD and healthy controls, and Haxby for decoding eight categories of visual objects. Final sample sizes, class composition, and demographic statistics should be based on the quality-controlled analysis cohorts rather than the original repository sizes.

ABIDE integrates multi-site resting-state fMRI and phenotypic information. We used ASD and healthy-control samples that met the quality-control criteria and task definition. The source repository contains 1,112 participants; the final sample size, class ratio, age distribution, and sex distribution should be reported for the quality-controlled analysis cohort.

ADHD-200 provides multi-site resting-state fMRI data from participants with ADHD and healthy controls. The source repository contains 776 participants; the final sample size, class ratio, and demographic statistics should likewise be reported for the quality-controlled analysis cohort.

Haxby is a task-based fMRI dataset for visual object recognition that includes six healthy participants and eight object categories. We used this dataset to evaluate multiclass brain-activity decoding rather than disease-vs.-control classification. Validation should group samples by participant, scan run, or stimulus block to prevent correlated observations from the same participant from appearing in both training and test sets.For all experimental analyses in this study, sample sizes, compositional categories, and demographic characteristics are based on the final analysis cohort --which underwent rigorous quality control --rather than the initial size of the raw database; a summary of task types, experimental paradigms, and baseline information for the verified post-quality-control cohorts is presented in Table 1.

**Table 1:** Dataset tasks and currently verifiable cohort information.

Dataset	Task	Acquisition	Public repository size	Reporting status of the analysis cohort
ABIDE	ASD vs. healthy controls	Resting-state, multi-site	1,112 participants	Final QC sample size and demographics to be reported
ADHD-200	ADHD vs. healthy controls	Resting-state, multi-site	776 participants	Final QC sample size and demographics to be reported
Haxby	Eight-class visual decoding	Task-based	Six healthy participants	Class counts and grouped splitting to be reported

### 3.2 Multi-site data harmonization

ABIDE and ADHD-200 comprise data from multiple acquisition sites. Differences in scanners, sequences, and acquisition parameters can introduce site effects into functional connectivity features that are unrelated to disease. We first calculated Pearson correlation coefficients between regional time series to obtain a symmetric 90×90 functional connectivity matrix for each participant, then vectorized its upper-triangular elements. neuroCombat (35) treated acquisition site as the batch variable and diagnostic category, age, and sex as biological covariates. Its empirical Bayes estimates corrected site-related additive and multiplicative effects. The harmonized vectors were then reconstructed as symmetric matrices for simplicial-complex construction.

1. Feature vectorization: Pearson correlation coefficients between brain regions of subjects were calculated to generate a 90×90 original functional connectivity matrix. To perform batch correction using neuroCombat, the upper triangular part of the matrix was extracted and vectorized to form a global feature matrix with dimensions of number of subjects × number of features. Preliminary statistical analysis revealed significant heterogeneity within the original multicenter dataset. As shown in Figures 2a,d, the ABIDE and ADHD-200 datasets exhibited a significantly unbalanced distribution in terms of sample size across different acquisition sites. Nevertheless, the proportions of diagnostic categories (ASD/ADHD vs. HC) within each site remained relatively balanced, which laid a data foundation for subsequent cross-center studies.
2. Batch effect modeling: Acquisition sites were used as batch variables, and subject diagnostic categories (ASD vs. HC), age, and gender were included as covariates. By modeling the mean (additive effect) and variance (multiplicative effect) of each site, neuroCombat removed site-specific biases while maximizing the retention of key biological variations.
3. Post-correction data restoration: The harmonized feature vectors were remapped into 90×90 symmetric matrices, which were used as inputs for the subsequent TSCNet model.

Figure 2 presents a two-dimensional PCA visualization of connectivity features before and after harmonization. After harmonization, samples from different sites showed greater overlap in the space of the first two principal components, indicating a reduction in visible site separation. However, PCA visualization cannot establish that all site effects have been removed or that disease-related variation has been fully preserved. Model performance must therefore be assessed using independent test folds under a rigorous cross-validation protocol.

**Figure 2:**
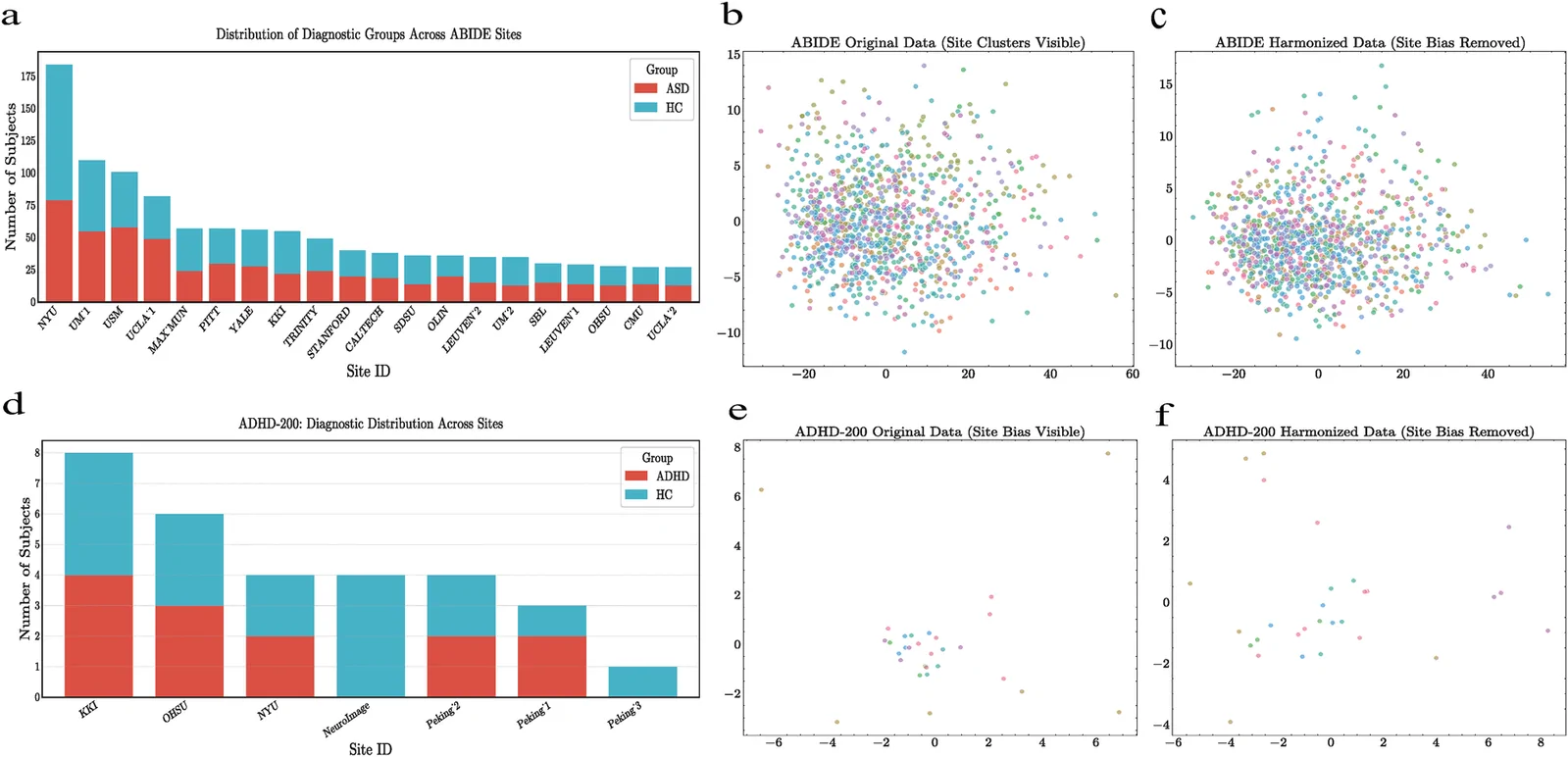
Site-wise class balance and harmonization of multi-center fMRI datasets. **(a)** Distribution of diagnostic groups across sites in the ABIDE dataset. Red: ASD patients; Blue: healthy controls (HC). **(b)** 2D embedding of raw functional connectivity data from ABIDE, showing prominent site-driven clustering. **(c)** ABIDE data after neuroCombat harmonization; site-related bias is removed and samples are well mixed. **(d)** Distribution of diagnostic groups across sites in the ADHD-200 dataset. Red: ADHD patients; Blue: healthy controls (HC). **(e)** 2D embedding of raw functional connectivity data from ADHD-200, showing visible site clustering. **(f)** ADHD-200 data after neuroCombat harmonization, with site-specific distributional shifts corrected.

Before correction (Figures 2b,e): Sample points exhibited an obvious trend of aggregation by acquisition site, indicating that hardware differences and acquisition parameters (e.g., magnetic field strength and scanning sequence) among different scanning centers introduced significant systematic interference to the data distribution, and such non-biological biases often masked the true neuropathological features.

After harmonization (Figures 2c,f), previously separated site clusters overlapped more strongly in feature space, and distributions from different centers became more similar. This pattern suggests that neuroCombat reduced visible between-site variation while retaining participant-level biological information. The harmonized matrices were subsequently used for higher-order topological feature extraction.

### 3.3 Simplicial complex construction

To go beyond traditional binary brain network representations and effectively capture high-order functional interactions of multi-region brain coordination, this study employs simplicial complex for hierarchical modeling of functional brain networks. First, the functional connectivity matrix R corrected by neuroCombat harmonization is converted into a topological distance matrix D, with the formula $D_{ij}=1-|R_{ij}|$, thereby mapping strong functional correlations to short topological distances. Subsequently, topological manifold extraction is performed on matrix D based on a preset filtering threshold ϵ (i.e., maximum edge length): a set of brain region nodes $\sigma =v_{0},\ldots \;v_{l}$ is defined as a k-dimensional simplicial complex if the distance between any two nodes in the set is no greater than ϵ.

This construction represents the functional brain network as a hierarchy of simplices: 0-simplices correspond to brain-region nodes, 1-simplices represent pairwise connections, and 2-simplices (triangular faces) represent the smallest group unit formed by three brain regions. We set the maximum simplex dimension to 2 to balance computational cost against the capacity to capture higher-order features, thereby providing multi-order local topological inputs to TSCNet. Sensitivity analysis indicated that performance peaked around ϵ=0.45.

During implementation, 0-, 1-, and 2-simplices form sets of nodes, edges, and triangles, respectively. Boundary matrix $B_{1}$ describes oriented node-edge incidence, whereas $B_{2}$ describes oriented edge-triangle incidence. The resulting $L_{0}$, $L_{1}$, and $L_{2}$ encode within-order topological adjacency in the node, edge, and triangle domains. Boundary matrices and Hodge Laplacians are stored sparsely to avoid dense computation over nonexistent higher-order relationships. Importantly, each 2-simplex in this study is formed by the closure of three pairwise correlation edges. It is a joint topological unit involving three brain regions, not an irreducible three-variable causal interaction.

### 3.4 TSCNet framework

The TSCNet forward pass comprises three parallel within-order branches and a cross-order global fusion module. For each participant, the functional connectivity matrix generates sets of 0-, 1-, and 2-simplices together with $L_{0}$, $L_{1}$, and $L_{2}$. The three SCNN branches separately produce node features $H_{0}$, edge features $H_{1}$, and triangle features $H_{2}$; the architecture does not sequentially transform 0-order features into 1- and 2-order features. Outputs from all orders are projected into a shared embedding space while retaining their order identity, arranged as an input sequence, and processed by multi-head self-attention to model dependencies across positions and orders. A readout operation and classifier then produce binary predictions for ABIDE and ADHD-200 or visual-category predictions for Haxby. Detailed reporting of SCNN channels, filter order, attention heads, embedding dimension, sequence masks, and classification readout is required for complete implementation-level reproducibility. Figure 3 illustrates the overall data flow, multi-stage branching structure, and cross-stage global fusion framework of TSCNet.

**Figure 3:**
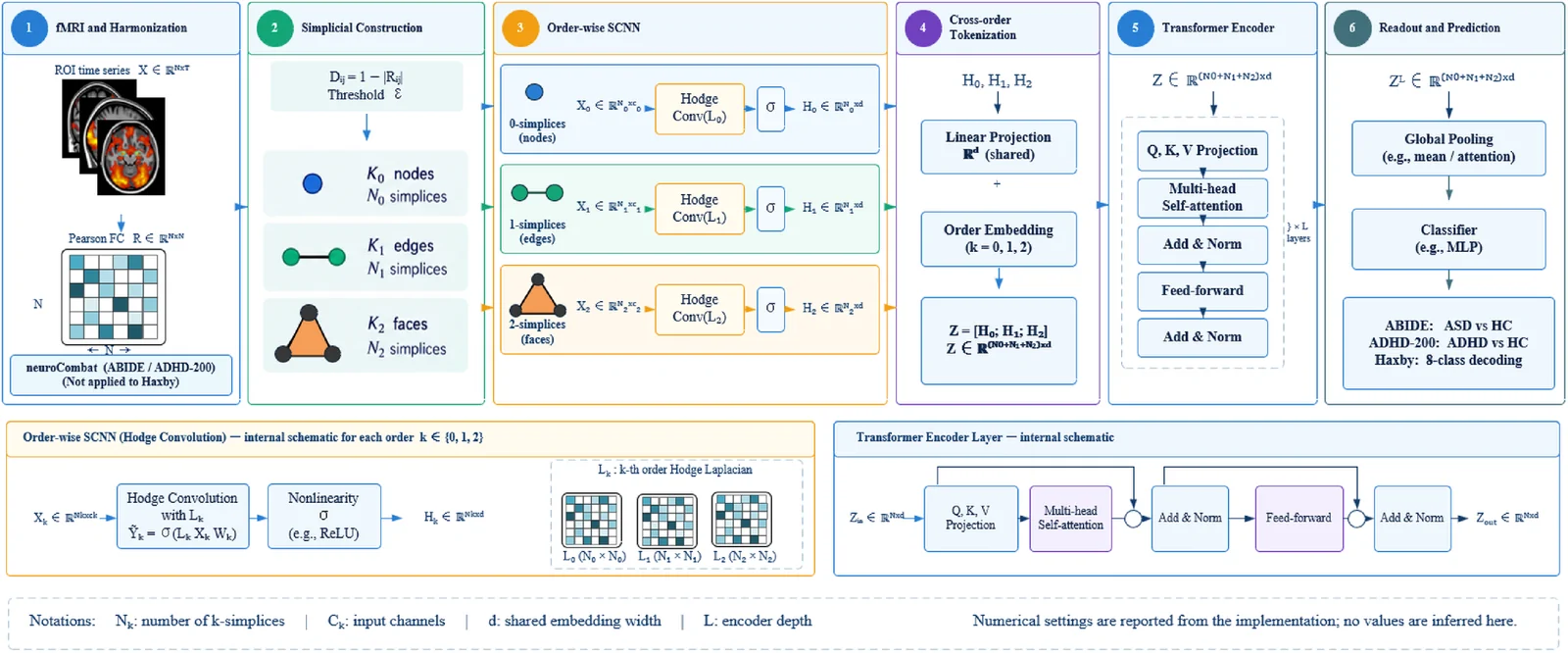
TSCNet data flow and cross-order fusion. Connectivity features from ABIDE and ADHD-200 are harmonized with neuroCombat and used to construct simplicial complexes up to order 2. $L_{0}$, $L_{1}$, and $L_{2}$ drive the 0-, 1-, and 2-order SCNN branches, respectively. The uniformly projected multi-order representations are processed by a Transformer to model global dependencies for binary disease classification or Haxby visual-category decoding.

The model extracts local topological features $H_{k}$of simplexes of each order through SCNN. For the input simplex signal $s^{k}$ of k order, its convolution extraction process is as follows in Equation 7:

$$H_{k}=\sigma \left(\sum_{\;p=0}^{P}\theta_{p}L_{k}^{p}S^{k}\right),$$ (7)

where $L_{k}$ is the k order Hodge Laplacian operator, $\theta_{p}$is the learnable filter coefficient, and p is the order of the convolution kernel. This module effectively captures the local topological associations from nodes and edges to higher-order surface units in the brain network through the Hodge Laplacian operator. On this basis, the model combines the multi-head attention mechanism of Transformer to achieve dynamic integration of multi-scale features. First, the features of each order are concatenated and mapped into a unified embedding vector $H_{in}$, and global dependencies in different subspaces are captured in parallel by multiple independent attention heads represented as Equation 8:

$${\mathrm{head}}_{i}=\mathrm{Attention}\left(H_{\mathrm{in}}W_{i}^{Q},H_{\mathrm{in}}W_{i}^{K},H_{\mathrm{in}}W_{i}^{V}\right),$$ (8)

where $W_{i}^{Q}$, $W_{i}^{K}$, and $W_{i}^{V}$ are the learnable linear projection matrices of the attention head i. Then, the global information extracted by each head is aggregated using a concatenation operation represented as Equation 9:

$$H_{\mathrm{global}}=\mathrm{Concat}\left({\mathrm{head}}_{1},{\mathrm{head}}_{2},\ldots ,{\mathrm{head}}_{h}\right).$$ (9)

This process enables the model to transcend the local receptive field of SCNN and dynamically identify long-range functional interaction patterns between different simplex orders at the whole-brain scale. Finally, the fused feature vector is input into a classifier to achieve accurate classification of brain diseases and functional localization of brain regions. The framework performance is systematically evaluated using multi-dimensional metrics such as accuracy and AUROC.

## 4 Results and discussion

### 4.1 Experiment settings

All algorithms in this study were implemented based on the Python 3.8 environment, with core topological computations and deep learning frameworks relying on the GUDHI, Nilearn, and PyTorch libraries, respectively. Model training was completed on a hardware platform equipped with an NVIDIA RTX 4060 GPU and 64 GB of memory, and the Adam optimizer was uniformly adopted for parameter updates. To reduce variability caused by a single split or random initialization, we used stratified 10-fold cross-validation and repeated the complete experiment independently five times. All classification metrics should be reported as mean ± standard deviation, and model comparisons should use identical data splits and random seeds. Data-dependent harmonization, standardization, and hyperparameter selection must be fitted only on each training fold and then applied to the corresponding validation or test fold to prevent information leakage. Because Haxby includes only six healthy participants, its splitting unit and within-participant grouping constraints require separate specification.

### 4.2 Baseline comparison

We evaluated TSCNet using Accuracy, AUROC, and Sensitivity and compared it with BrainNetTF (14), FBNetGen (15), BrainNetCNN (16), and Graphormer (30). Figure 4 presents the overall distributions available in the archived experiments. Dataset-specific means and standard deviations for every method and paired statistical comparisons were not available in the current records; therefore, the following conclusions are limited to the plotted trends and verifiable point estimates. To clearly and systematically outline the verifiable experimental data, missing baseline results, and limitations of the research evidence, Table 2 summarizes the task configurations, verifiable experimental results for TSCNet, the status of existing baseline results, and the boundaries of the evidence for each dataset.

**Figure 4:**
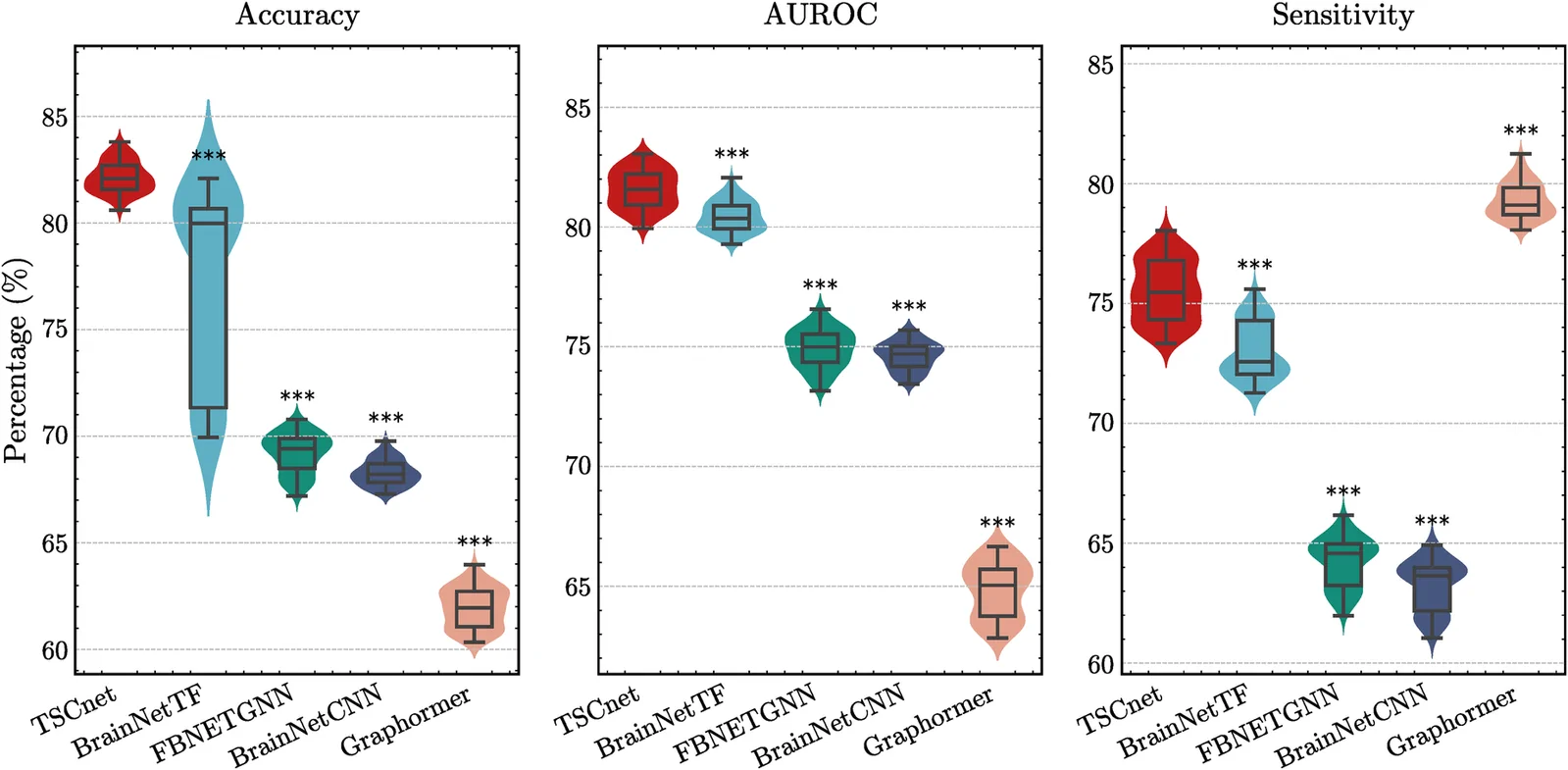
Overall comparison of model-performance distributions. *** indicates p<0.001; the statistical unit and correction procedure should be specified using the original repeated-experiment records.

**Table 2:** Summary of verifiable dataset-specific results.

Dataset	Task	Verifiable TSCNet result	Other available evidence	Evidence boundary
ABIDE	ASD/HC binary classification	Accuracy 81.23%	Ablations and Figure 4	Missing per-baseline mean ± SD
ADHD-200	ADHD/HC binary classification	Accuracy 82.42%	Ablations and Figure 4	Missing per-baseline mean ± SD
Haxby	Eight-class visual decoding	Sensitivity distribution	Execution time 0.05 min	Missing Accuracy, AUROC, and exact mean ± SD

Figure 4 shows that TSCNet has relatively high overall distributions for Accuracy and AUROC, whereas the relative ranking of models is less consistent for Sensitivity. The current evidence therefore suggests a favorable, but not uniformly dominant, trade-off between local higher-order topology and global attention across metrics. Without a complete dataset-specific statistical table and reproducible test details, the results should not be generalized as significant superiority over every baseline on every metric and dataset.

The Haxby experiment evaluates decoding of eight visual-object categories in healthy participants rather than brain disorder diagnosis. The available figure supports only a distributional comparison of Sensitivity and does not report Accuracy, AUROC, macro-Sensitivity, confusion matrices, or cross-participant results. Haxby therefore demonstrates that the framework supports multiclass output, but it does not directly establish disease-subtype recognition or clinical multiclass capability.

### 4.3 Ablation study

On ABIDE, the complete TSCNet achieved an Accuracy of 81.23%. Removing the SCNN reduced Accuracy to 78.36%, a decrease of 2.87 percentage points, while removing the Transformer reduced it to 75.34%, a decrease of 5.89 points. The corresponding values on ADHD-200 were 82.42%, 79.42%, and 76.29%, representing decreases of 3.00 and 6.13 points. The consistent pattern across both datasets indicates that the local topological information provided by the SCNN and the global dependency modeling provided by the Transformer each contribute to classification. Because Figure 5 reports point estimates without an interaction control, these results support complementary contributions but do not by themselves establish a statistically significant synergistic effect. To intuitively illustrate the specific performance changes resulting from the ablation of various modules and to clearly summarize the verifiable point estimates from ablation experiments across both datasets, this study has compiled a summary of the quantitative results of the TSCNet module ablation, as presented in Table 3.

**Figure 5:**
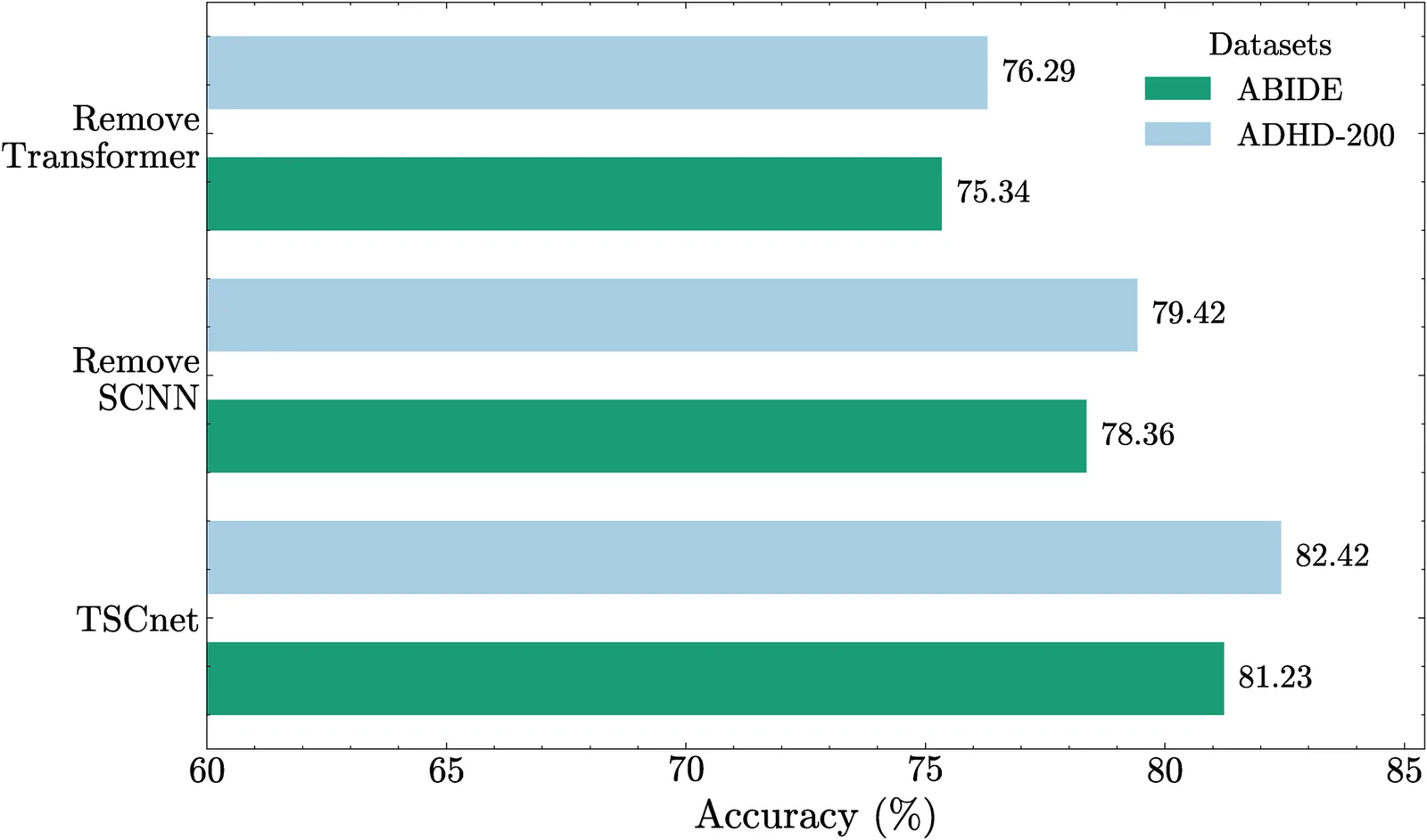
Ablation experiment.

**Table 3:** Verifiable point estimates from the TSCNet module ablation.

Model variant	ABIDE accuracy	Decrease	ADHD-200 accuracy	Decrease
TSCNet	81.23%	–	82.42%	–
Without SCNN	78.36%	2.87 pp	79.42%	3.00 pp
Without Transformer	75.34%	5.89 pp	76.29%	6.13 pp

### 4.4 Sensitivity analysis

To investigate the robustness of the TSCNet model under different parameter configurations and quantitatively analyze the contribution of high-order topological representations to classification performance, a systematic sensitivity analysis was conducted for the filtering threshold ϵ, embedding dimension d, simplex order (Order), and number of network layers L, with the results shown in Figure 6.

**Figure 6:**
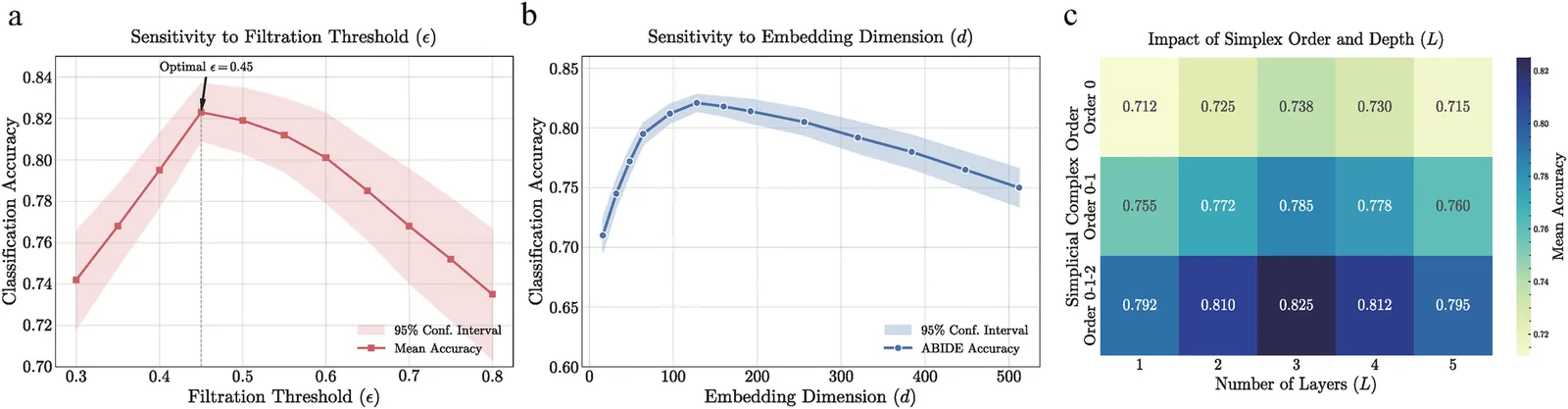
Parameter sensitivity analysis.

The filtration threshold ϵ determines which functional connections enter the simplicial complex. Because $D_{ij}=1-|R_{ij}|$, increasing ϵ admits weaker correlations and increases the numbers of edges and 2-simplices. A small ϵ can produce an overly sparse complex and omit potential coordinated structures, whereas a large ϵ may close noisy correlations into many triangular faces. Figure 6a shows that Accuracy first increased and then decreased as ϵ rose from 0.3 to 0.8, peaking at 0.45. The relatively high performance at neighboring values indicates that the model is not effective only at an isolated threshold. Nevertheless, candidate brain-region combinations require further validation using cross-threshold overlap and stability across repeated experiments.

Figure 6b shows that increasing the embedding dimension from 16 to 128 improved Accuracy from 71.0% to 82.1%, whereas further increasing it to 512 reduced Accuracy by approximately 7%. This pattern indicates a trade-off between representational capacity and overfitting in a small-sample setting. The curve supports 128 as a favorable choice within the current search range, but not as a universally optimal value for other datasets.

Figure 6c shows that, at the same network depth, extending simplex order from 0 only to 0-1-2 increased the highest Accuracy from 73.8% to 82.5%, with the best result under the current setting obtained at a depth of three layers. This finding supports the incremental value of 2-simplex information, but it does not imply that every highly weighted triangular face corresponds to a real and independent biological process.

### 4.5 Comparison of functional connectivity in brain regions

To visually compare the ability of different models to capture functional brain network features, this study uses the ABIDE dataset as the object and shows the differences between TSCNet and BrainNetTF in brain region activation patterns from the perspective of high-order topological features of brain functional networks. The results are shown in Figure 7.

**Figure 7:**
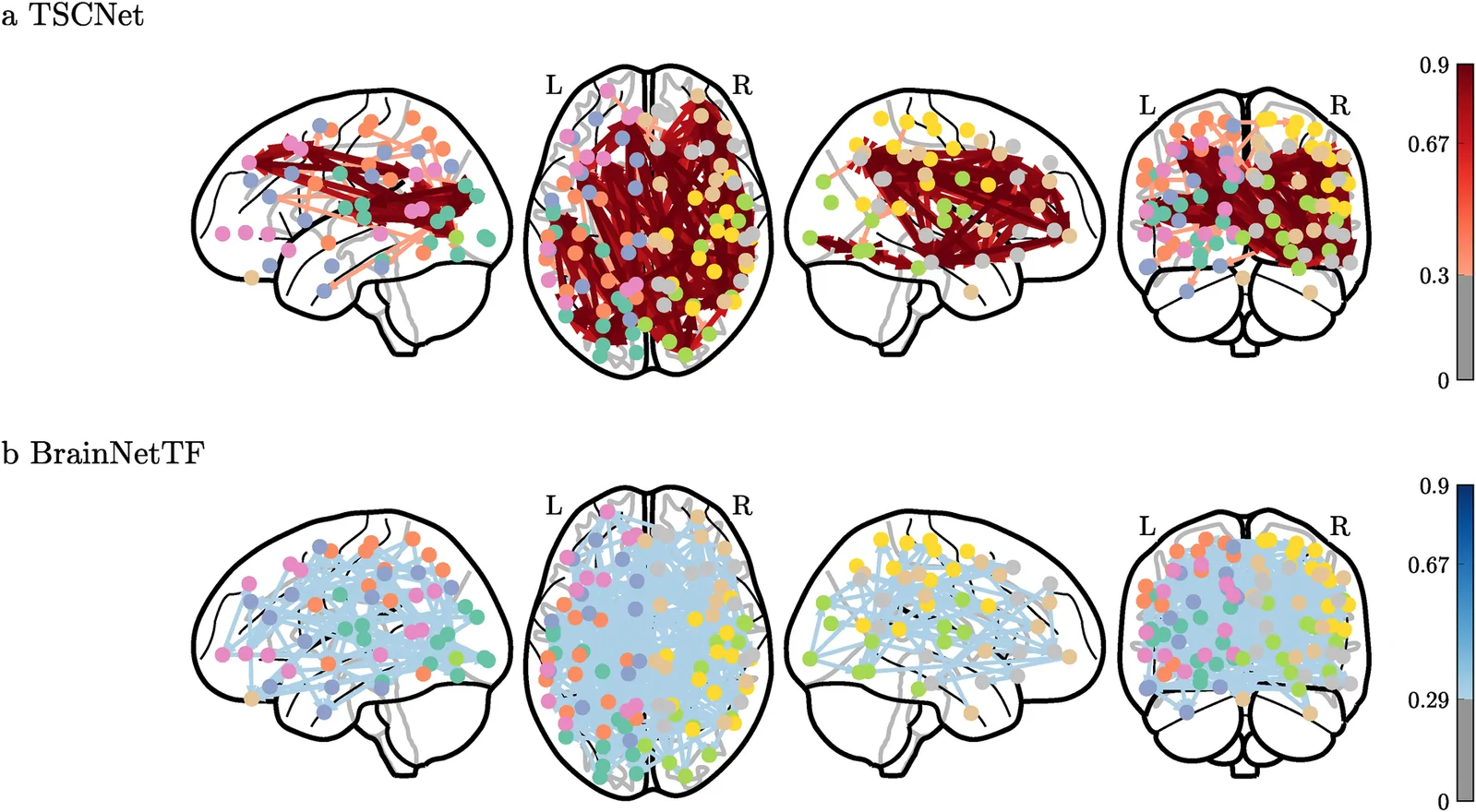
Multi-perspective and intensity comparison of functional connectivity in brain regions. **(a)** TSCNet: hierarchical visualization using light red 1-simplex (representing the strength of binary connectivity between brain regions) and dark red 2-simplex (representing the higher-order topological strength of triangular synergy among multiple brain regions) on a red scale (0-0.9, with a threshold of 0.3); **(b)** BrainNetTF: presentation of the strength of paired associations between brain regions using light blue binary connectivity on a blue scale (0-0.9, with a threshold of 0.29).

In the thresholded complex used here, a 2-simplex indicates that all three pairwise functional connections among three brain regions pass the threshold and form a closed triangle. It contains richer group-level topology than a single edge, but it is not equivalent to an irreducible three-variable statistical interaction and cannot be directly interpreted as an anatomical polysynaptic pathway, direction of information flow, or causal coordination. Figure 7 should therefore be viewed as a visualization of candidate higher-order functional coordination patterns rather than direct evidence of neural-circuit mechanisms. If verified regional labels show that highly weighted triangles involve the default mode, frontoparietal control, or sensorimotor networks, they may be related to previous findings on altered cross-network integration in ASD (4, 36). However, effect sizes between groups and cross-fold stability remain to be established, so deterministic terms such as precise correspondence or key biomarker are not warranted.

BrainNetTF primarily represents the brain network through pairwise connections, whereas TSCNet additionally retains thresholded triangular closures. This distinction explains the different levels of information shown in the two visualizations. Figure 7 is based only on ABIDE and should not be directly extrapolated as a neurobiological finding for ADHD. ADHD-related interpretations require stable 2-simplices extracted independently from ADHD-200, followed by tests of case-control differences, cross-site consistency, and symptom associations.

### 4.6 Computational efficiency

Figure 8 compares execution time under the same hardware environment rather than asymptotic computational complexity. On ABIDE, TSCNet required 2.85 min, compared with 22.40 min for FBNetGen and 153.50 min for Graphormer, indicating lower training-time overhead under the current implementation, input scale, and hardware. Possible reasons include sparse local operations on the simplicial complex and global attention in a reduced-dimensional embedding space. Execution time depends on implementation optimization, batch size, training epochs, and hardware. Clinical deployment will additionally require separate reporting of per-case preprocessing time, inference latency, peak GPU memory, parameter count, and simplicial-complex construction cost.

**Figure 8:**
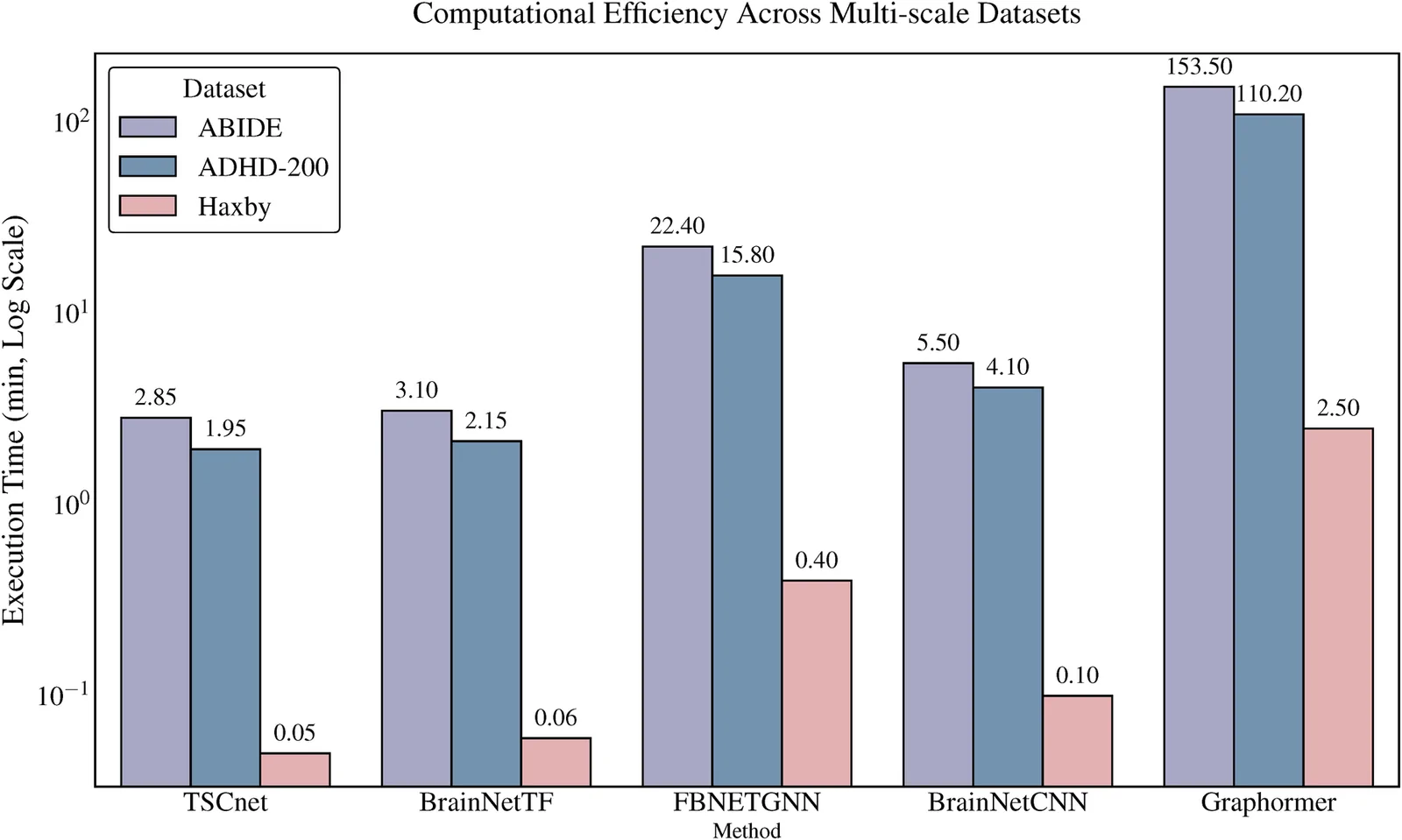
Comparison of execution time.

### 4.7 Robustness, clinical deployment, and study limitations

ABIDE and ADHD-200 are multi-site datasets, but cross-validation with mixed sites does not establish external generalization to unseen scanners or centers. Magnetic field strength, sequence parameters, head-motion distributions, recruitment criteria, and demographic composition can all produce domain shifts. neuroCombat can reduce batch differences among known sites, but adaptation to a new site still requires leave-one-site-out validation, temporal validation, and evaluation in independent clinical cohorts.

Medical imaging cohorts are often small and class-imbalanced, and complex models may learn site or demographic confounders. Before clinical deployment, quality control, missing-data handling, failure fallback, and uncertainty calibration should be prespecified, and fairness should be evaluated across subgroups defined by age, sex, head motion, and comorbidity. The current model output is better suited as auxiliary information than as an independent diagnostic conclusion.

Haxby shows that the framework can formally support multiclass output, but it does not establish clinical subtype-recognition capability. Disease-subtype analysis must also address inconsistent labels, overlapping subtype boundaries, long-tailed classes, small samples, and comorbidity. Future studies could use hierarchical classification, class reweighting, or metric learning, together with nested cross-validation and independent cohorts, and report macro-F1, balanced accuracy, macro-AUROC, and recall for each class.

Extending TSCNet to structural MRI and diffusion tensor imaging could introduce complementary information about brain morphology and white-matter structural connectivity, but would require cross-modal registration, alignment of feature scales, and handling of missing modalities. For phenotypes such as symptom severity and cognitive scores, feature selection should be performed only within the training data, and correlations, confidence intervals, and multiple-comparison corrections should be reported on independent test data. Only higher-order features that remain stable across folds and sites should be considered candidate imaging biomarkers.

## 5 Conclusion

We proposed TSCNet, which uses simplicial convolutional neural networks to encode local topological relationships among 0-order nodes, 1-order edges, and 2-order triangular faces, and a Transformer to integrate long-range and cross-order dependencies. This design extends conventional graph models that explicitly represent only pairwise connections. In the available experiments, TSCNet showed relatively high overall Accuracy and AUROC distributions in Figure 4. Removing either the SCNN or Transformer reduced Accuracy on ABIDE and ADHD-200, and parameter analysis showed that representations containing 0-, 1-, and 2-simplices outperformed those using only 0-order features. Together, these findings support the complementary value of local higher-order topological encoding and global dependency modeling.

This study has several limitations. The simplicial complex is constructed using a fixed threshold, and threshold variation affects topological density, classification performance, and candidate brain-region combinations. The ABIDE and ADHD-200 results are based on public retrospective data and have not yet been validated on unseen centers, unseen scanners, or prospective clinical cohorts. Haxby is a visual-object decoding task in healthy participants and does not directly demonstrate validity for disease-subtype classification. The current 2-simplex visualizations also require regional-label verification, cross-fold stability analysis, group-level statistics, and phenotypic association analysis before their value as biomarkers can be assessed.

Future work will improve the robustness of higher-order features through cross-threshold stability selection and data-driven thresholding; evaluate domain-shift performance using leave-one-site-out validation, independent cohorts, and uncertainty calibration; establish multiclass protocols for clinical subtypes under small-sample and class-imbalanced conditions; and integrate structural MRI, diffusion tensor imaging, and clinical phenotypes to test the incremental explanatory value of higher-order topology under strict independent validation and correction for multiple comparisons.

## Data Availability

ABIDE, ADHD-200, and Haxby are publicly available de-identified datasets that can be accessed via the links below. The source code required to reproduce the reported results can be found at https://github.com/922397935/TSCNet_main_v1/tree/main. The ABIDE dataset: The Autism Brain Imaging Data Exchange (ABIDE) dataset (containing 1112 anonymized neuroimaging samples from 16-17 international sites) is accessible through the Preprocessed Connectomes Project (PCP) repository: https://preprocessed-connectomes-project.org/abide/. This dataset contains structural/resting-state functional magnetic resonance imaging data and phenotypic information; all protected health information has been removed. The ADHD-200 dataset: The ADHD-200 dataset (containing 776 unrestricted samples from 8 imaging centers, including fMRI/anatomical data and phenotypic metadata) is available through the Neuro Bureau project on NITRC (http://www.nitrc.org/projects/neurobureau) and Amazon S3 buckets (http://preprocessed-connectomes-project.org/adhd200/download.html). Access to this dataset requires a registered NITRC account and adherence to non-commercial attribution requirements. The Haxby dataset: The Haxby dataset (neural response data to natural stimuli organized according to the BIDS standard) is hosted on OpenNeuro (dataset ID: ds000233; https://openneuro.org/datasets/ds000233 and the Datalad platform (http://datasets.datalad.org/?dir=/labs/haxby/attention). It can be directly accessed through neuroimaging tools (such as Nilearn) without any access restrictions.
